# Gaze onsets during naturalistic infant-caregiver interaction associate with ‘sender’ but not ‘receiver’ neural responses, and do not lead to changes in inter-brain synchrony

**DOI:** 10.1038/s41598-023-28988-0

**Published:** 2023-03-02

**Authors:** I. Marriott Haresign, E. A. M. Phillips, M. Whitehorn, F. Lamagna, M. Eliano, L. Goupil, E. J. H. Jones, S. V. Wass

**Affiliations:** 1 Department of Psychology, University of East London, London, E15 4LZ UK; 2 LPNC/CNRS, Grenoble Alpes University, Grenoble, France; 3 Centre for Brain and Cognitive Development, Birkbeck College, University of London, London, UK

**Keywords:** Psychology, Human behaviour

## Abstract

Temporal coordination during infant-caregiver social interaction is thought to be crucial for supporting early language acquisition and cognitive development. Despite a growing prevalence of theories suggesting that increased inter-brain synchrony associates with many key aspects of social interactions such as mutual gaze, little is known about how this arises during development. Here, we investigated the role of mutual gaze *onsets* as a potential driver of inter-brain synchrony. We extracted dual EEG activity around naturally occurring gaze onsets during infant-caregiver social interactions in N = 55 dyads (mean age 12 months). We differentiated between two types of gaze onset, depending on each partners’ role. ‘Sender’ gaze onsets were defined at a time when either the adult or the infant made a gaze shift towards their partner at a time when their partner was either already looking at them (mutual) or not looking at them (non-mutual). ‘Receiver’ gaze onsets were defined at a time when their partner made a gaze shift towards them at a time when either the adult or the infant was already looking at their partner (mutual) or not (non-mutual). Contrary to our hypothesis we found that, during a naturalistic interaction, both mutual and non-mutual gaze onsets were associated with changes in the sender, but not the receiver’s brain activity and were not associated with increases in inter-brain synchrony above baseline. Further, we found that mutual, compared to non-mutual gaze onsets were not associated with increased inter brain synchrony. Overall, our results suggest that the effects of mutual gaze are strongest at the intra-brain level, in the ‘sender’ but not the ‘receiver’ of the mutual gaze.

## Introduction

Most of our early life is spent in the presence of an adult social partner. Most early attention—and, in particular, most early cognitive learning—takes place in social settings^1^. But almost all of our understanding of how the brain subserves attention and learning has come from studies that measure individual brains in isolation.

In recent years our understanding of early social and communicative development has relied heavily on studying ostensive signals, defined as signals from a communicator to generate an interpretation of communicative intention in an addressee. It has been argued that, from shortly after birth, infants’ brains are sensitive to ostensive signals (such as direct gaze, smiles and infant-directed vocalisations), and that ‘sender’ communicative signals play a key role in supporting early learning exchanges^1–7^. In this paper we focus on mutual gaze, which is a widely studied ostensive signal.

Farroni et al.^8^ found that images of faces showing direct versus averted eye contact elicited greater amplitude event related potentials (ERPs) in infants even 2- to 5-days after birth. Grossman et al.^9^ observed greater gamma power activation in 4-month-old infants in response to facial stimuli with direct gaze versus averted gaze, and where an experimenter engages in mutual gaze before looking towards an object, infants show enhanced neural processing of those objects^10,11^. Of note, findings of gaze orientation on ERP amplitudes have not replicated well in developmental research; for example, Elsabbagh et al.^12^ found no significant effect of gaze type, comparing ERP amplitudes (see control group comparison); and findings are also largely mixed in adults (e.g.^13–18^). The above research with infants and adults converges that neural responses to eye contact typically happen rapidly and transiently, with the first deflections in amplitude peaking within 100–200 ms of stimulus presentation and returning to baseline after 500–600 ms. Despite the inconsistencies, these findings have contributed to the popular idea that infants are highly sensitive to their partner’s social signals during early learning exchanges^2,19–23^, see Cetincelik et al.^24^ for a review. This raises basic questions of in what contexts, and under what circumstances are infants sensitive to their partner’s social signals.

One important limitation of research measuring infants’ neural responses to social stimuli presented on a screen, however, is its limited ecological validity. Historically, the majority of studies into early social development have measured infants’ passive responses to viewing a series of static images that flash on and off on a screen in a predetermined sequence. The real world, in contrast, is interactive, contingent and continuous. In recent years an increasing number of researchers have begun to recognise that, to study how the infant brain subserves social interaction, it is necessary to actually study it in interactive contexts^25–28^. Recent behavioural findings have suggested important differences between screen-based simulacra of social interaction and actual social interaction. For example, recent studies^29,30^ have shown that infants rarely look to their caregiver’s face and eyes during free-flowing interactions, this is in contrast to what has previously been shown using screen-based tasks (e.g.^21,31^). So far, the neural processing of mutual gaze has largely been investigated in un-ecological contexts, and in the absence of real social interaction. Consequently, two important questions remain unanswered; (1) is mutual gaze really a salient communicative signal during free-flowing social interactions occurring in rich, continuous, natural scenes? (2) how does intra and inter-brain activity support the processing of sender and receiver ostensive signals such as mutual gaze when both partners are engaged in a free-flowing, bidirectional exchange of information?

### Mutual gaze and inter-brain synchrony

Another topic that has shown rapidly burgeoning popularity in recent years is inter-brain synchrony. At the neural level, inter-brain synchrony can be defined as a dyadic mechanism, wherein temporally coordinated patterns of brain activity between two interacting individuals supports aspects of their ongoing social interaction^32^. A number of studies have observed increased inter-brain synchrony during mutual gaze. The majority of this research claims to measure inter-brain synchrony, although we recognise that not all of these studies will meet the framework of inter-brain synchrony set out in more recent theoretical accounts^32^. Kinreich and colleagues^33^ observed significantly correlated gamma (30–60 Hz) activity between interacting adults during social interaction. Higher interpersonal gamma correlations were also associated more strongly with mutual versus non-mutual gaze. Similarly, Luft and colleagues^34^ found that mutual gaze was associated with higher inter-brain gamma band (30–45 Hz) coherence (a spectral measure based on correlation) between interacting adults than non-mutual gaze. In the developmental literature, our group investigated inter-brain synchrony in 7.5-month infant-adult dyads during moments of mutual and non-mutual gaze^35^. During a live social, but not interactional condition infants observed an adult singing nursery rhymes, who was instructed to look either directly at the infant, directly at the infant with their head turned at a slight angle, or away from the infant. Consistent with research on adults, we found greater infant-adult neural synchrony during moments of mutual versus non-mutual gaze, measured using partially directed coherence (PDC-a spectral Granger causal measure of synchrony) in Theta (3–6 Hz) and Alpha (6–9 Hz) band activity. This study thus suggests that the impact of mutual gaze on inter-brain synchrony found in adult-adult dyads^33,34^ is already present early on in development, though possibly in lower frequency brain rhythms. Additionally, associations between mutual gaze and adult-infant inter-brain synchrony have been found using fNIRS^36^. However, because the temporal resolution is so different between EEG and fNIRS it is difficult to conclude that these represent the same neural mechanisms.

### Sender/receiver mechanisms of inter-brain synchrony

As recent theoretical accounts have highlighted^32,37,38^ inter-brain synchrony can reflect underlying mechanisms of varying complexity. To date, research investigating inter-brain synchrony during social interaction has exclusively measured this using non-event locked analyses, i.e., inter-brain synchrony values are averaged across whole conditions and/ or whole interactions and not time locked to any specific events within the interaction. Previously we have argued that in order to distinguish between different mechanisms that might give rise to inter-brain synchrony it is important to use event locked analyses^39^, i.e., analyses that focus on measuring fine-grained temporal changes in inter-brain synchrony, time-locked to specific behaviours/ events within social interactions. Additionally, when trying to differentiate inter-brain synchrony from other forms of inter-personal neural synchrony, it is also important to measure dyadic dynamics, e.g., how both partners’ behavior and neural activity contribute to establishing inter-brain synchrony.

We have suggested that one leading candidate mechanism for establishing inter-brain synchrony during mutual gaze may be mutual phase resetting^27,35^. It is known that the phase of neuronal oscillations reflects the excitability of underlying neuronal populations to incoming sensory stimulation^40,41^. Consequently, there has been much effort expended in recent years, across a range of research fields, on exploring whether neuronal oscillations could be a key mechanism for temporal sampling of the environment^42–46^. For example, some evidence suggests that sensory information arriving during high-receptivity periods is more likely to be perceived than information arriving during low-receptivity periods^47–51^. This suggests that there is an optimal (range of) phase for perceiving information. It has been argued that if this is correct then it is logical to assume that there exist mechanisms, either endogenous or exogenous, for modulating the phase of neuronal oscillations, in order to match the temporal structure of the environmental input^46,52^. This mechanism (phase resetting) would enable more efficient processing as information would be received during periods (phases) of high receptivity.

Empirical evidence supports the role of phase resetting as an intra-brain mechanism, facilitating neural entrainment to temporal structures within the environment, for example speech^43,53^. It is therefore logical to question whether similar mechanisms also operate at the interpersonal level. For example, inter-brain synchronisation may increase within a dyad following the onset of communicative signals (such as gaze, gestures, or vocalisations) that reset the phase of both interacting partners. Here, neural oscillations in both the sender (of the social signal) and the receiver’s brain that were previously random with respect to each other (low inter-brain synchrony) would be simultaneously reset in response to a communicative signal. Following this reset the neural activity of both the sender and the receiver would oscillate with more consistent variation over time (high inter-brain synchrony). We recognise that according to recent theoretical accounts that this might be classed as motor-induced neural synchrony, which occurs when the behaviour of one member of the dyad drives the neural activity of both members of the dyad^32^. However, it could also be that mutual gaze onsets reset the brain activity of the sender, which precedes/causes the behaviour of the receiver, which then causes a reset in the receiver’s brain activity. Here, increases in inter-brain synchrony would be a result of both partners resetting to their own behaviours. Distinguishing between these different mechanisms is only possible using event locked analysis. It is also worth noting here previous research that has examined the timescale of phase resetting in response to visual processing of images of faces presented on a screen. For example, Rousselet and colleagues^54^ found that in adults, changes in phase occurred rapidly and transiently in response to the presentation of faces, peaking and returning to baseline levels between 0 and 500 ms of stimulus presentation.”

### Eye movement (saccade) related potentials and ERPs

One challenge in studying the impact of mutual gaze during naturalistic social interaction on dyadic EEG is that mutual gaze onsets are time-locked to eye movements which create multiple types of artifact in the EEG^55^. For example, Plöchl and colleagues^56^ showed that saccadic spike potentials (EEG potentials time-locked to small, < 1°, involuntary eye movements during fixation) typically introduce a broadband artifact in the time–frequency spectrum of the EEG, which is strongest (amplitude) in the low beta (~ 14–30 Hz) and gamma bands (> 30 Hz) of adult EEG and typically peaks between − 50 ms and 150 ms around the offset of a saccade. Artifact generated from eye movements can overlap in time and frequency with EEG activity presumed to be related to genuine neural activity, associated with stimulation of the retina^57–59^. This is often referred to as the lambda response (LR)^60^, which is an occipital EEG potential that can be observed when saccades are made against an illuminated contrast background^61^. LRs typically produce broadband (0–50 + Hz) time–frequency activity that is strongest (amplitude) in Alpha (8–13 Hz) and low beta (~ 14–30 Hz), over occipital electrodes and peaks ~ 100 ms after the offset of the saccade ^62,63^. The overlapping activations introduced by eye movements can make interpretation of the data challenging, a problem which is not solved using ‘standard’ artifact correction procedures which fail to completely remove artifact associated with eye moments from the EEG; both in adults^54,55^ and infants^64^.

Our analyses are presented using a pipeline specially designed for the removal of eye movement artifact from naturalistic EEG data using ICA^64–66^. However, it is important to note that in our 2021^64^ paper we reported that we (as arguably most of the current research in developmental neuroscience using EEG is) were unable to completely remove the activity that we assumed to be artifactually related to eye movements. Therefore, in this current work, it is likely that the sender neural responses that we are investigating are a combination of some residual artifactual activity; although as discussed above these artifacts are transient (~ 100 ms) and therefore would only impact the initial part of the ERP waveform, and genuine neural activity; after the initial ~ + 150–200 ms. For this reason, our primary analyses will compare sections of our data that both contain saccades, and therefore have (we assume) an identical amount of eye movement artifact in them but have different consequences (either the saccade leads to mutual gaze, or not). Furthermore, we only compared activity in the later parts of the ERP waveform after the first 100 ms.

### Current study: the role of mutual gaze onsets in creating inter-brain synchrony

**Figure 1:**
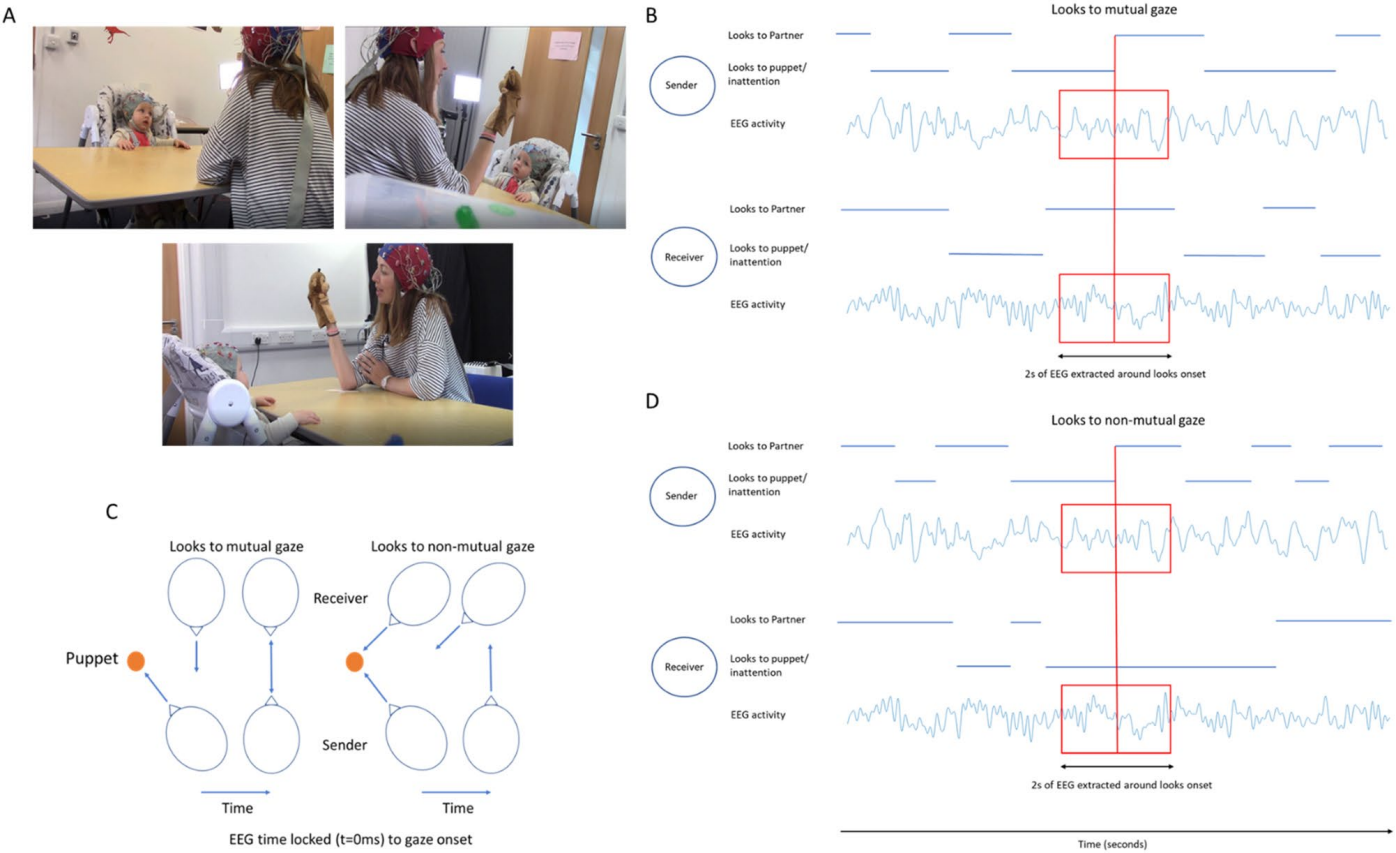
Illustration of experimental set-up and design for event locked analysis. (**A**) shows screenshots of experimental recordings from three camera angles used. (**B**) shows event locking process for sender and receiver EEG activity relative to mutual gaze onsets. Here receiver’s gaze was maintained on the sender and the sender’s gaze shifted from looking at the puppet/inattention toward looking at the receiver, facilitating mutual gaze. ± 1 s of EEG activity was extracted around gaze shift. (**C**) shows topographical illustration of sender’s and receiver’s gaze relative to mutual and non-mutual gaze onsets. (**D**) shows event locking process for sender and receiver EEG activity relative to mutual gaze onsets. Here receiver’s gaze was maintained on either the puppet (or inattention) and the sender’s gaze shifted from looking at the puppet/inattention toward looking at the receiver, facilitating non-mutual gaze. ± 1 s of EEG activity was extracted around gaze shift.

**Figure 2:**
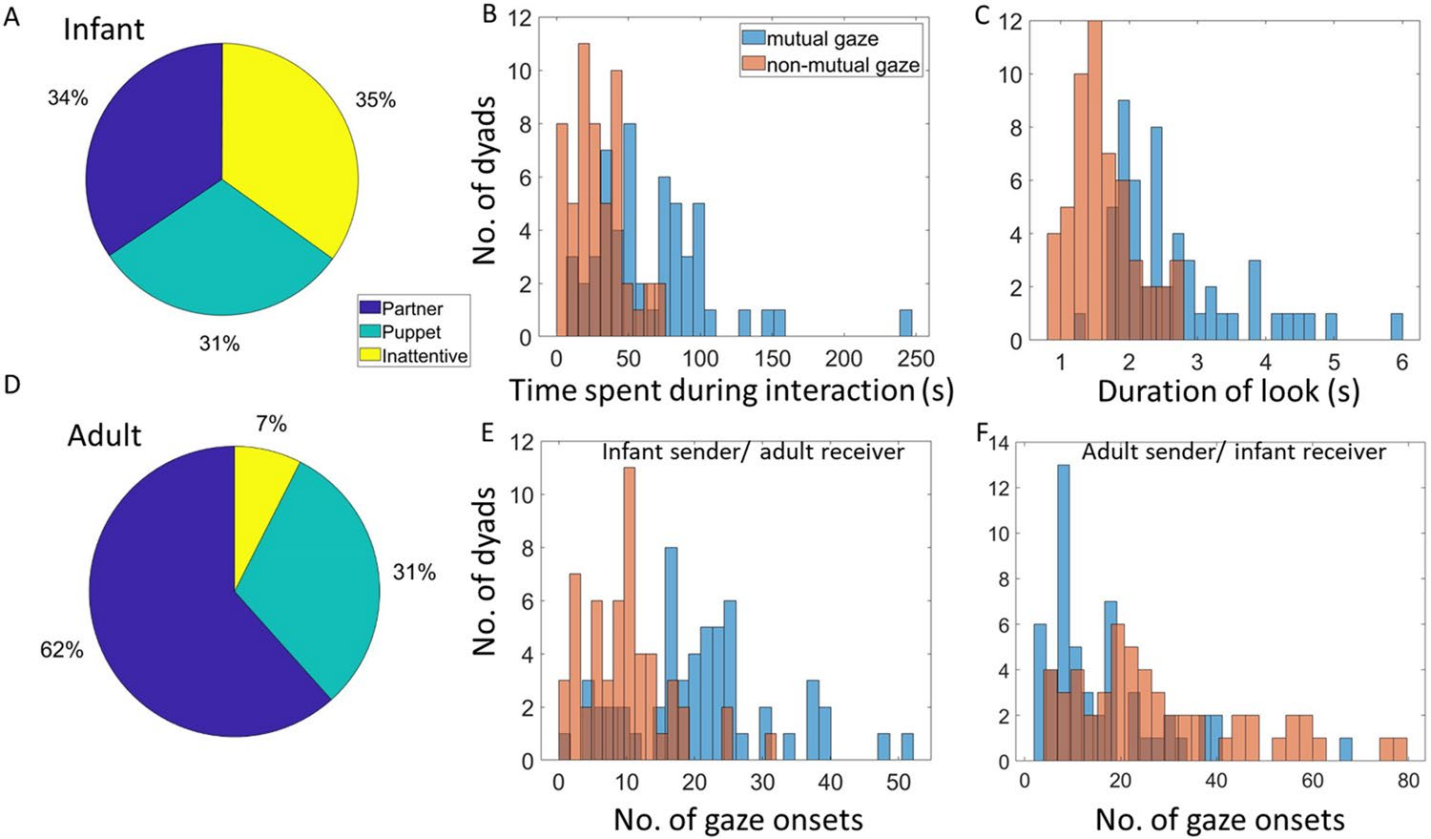
Distribution of gaze onsets in our sample. (**A**) Distribution of time infants spent looking at different areas. (**B**) Distribution of time dyads spend in mutual and non-mutual gaze during interaction. (**C**) Distribution of look durations for mutual and non-mutual gaze defined from infants’ look behaviour. (**D**) Distribution of time adults spent looking at different areas. (**E**) Distribution of infant sender/adult receiver mutual and non-mutual gaze onsets. (**F**) Distribution of adult sender/infant receiver mutual and non-mutual gaze onsets.

Our study aimed to test the hypothesis that infants are sensitive to ostensive signals during free-flowing social interactions, and that mutual gaze onsets lead to mutual phase resetting, which causes increases in inter-brain synchrony. We measured dual EEG recordings from parents and infants whilst they engaged in free-flowing social interactions and investigated intra- and inter- individual neural responses to naturally occurring moments of mutual gaze. To explore the role of turn-taking in creating inter-brain synchrony, we differentiated between two types of look onset, depending on each partners’ role. ‘Sender’ gaze onsets were defined at a time when either the adult or the infant made a gaze shift towards their partner at a time when their partner was either already looking at them (sender mutual) or not looking at them (sender non-mutual). ‘Receiver’ gaze onsets were defined at a time when their partner made a gaze shift towards them at a time when either the adult or the infant was already looking at their partner (receiver mutual) or not (receiver non-mutual) (see Fig. 1). For all of our analysis we wanted to explore how different aspects of the EEG signal were changing around behavioural events (mutual and non-mutual gaze onsets), therefore we needed to define clear points for which we were going to extract the EEG around. Obviously, this breaks the continuous nature of the data that we collected but we felt like the design of the current analyses was important for trying to investigate the mechanisms that might give rise to inter-brain synchrony in a temporally fine-grained way. We chose shifts in gaze as a natural event boundary that we could extract segments of EEG activity around. Our definitions of ‘sender’ and ‘receiver’ describe each partners role relative to one singular action—one individual’s shift in gaze (saccade) from either the puppet or a state of inattention to their partners’ gaze. That is to say that both sender and receiver onsets are time locked to the same behavioural event—the senders gaze shift. We chose the term ‘sender’ to describe the active agent and ‘receiver’ to describe the passive agent relative to the behavioural event (saccade to partner). Both sender and receiver onsets are time locked to the exact same time within the interaction—there is no lag between the time locking of the EEG between sender and receiver onsets. The distributions of sender and receiver mutual and non-mutual gaze onsets are given within Fig. 2 (E shows distributions of infant sender/adult receiver mutual and non-mutual gaze onsets and F shows distributions of adult sender/infant receiver mutual and non-mutual gaze onsets).

In order to assess how these interpersonal dynamics contributed to inter-brain synchrony we used two different measures of synchrony; First, we used a measure of concurrent synchrony, Phase locking value (PLV), that measures zero-lag, undirected synchrony. This measure would best capture changes in inter-brain synchrony that resulted from changes in both partners’ brains concurrently. We also used a measure of sequential synchrony, Partially directed coherence (PDC), that measures time lagged, directed synchrony. This would best capture changes in inter-brain synchrony that resulted from changes in one partner’s brain that forward predicted or lead to changes in the other partner’s brain. Through this we were able to consider three main sets of research questions.

### Inter-brain non-event locked analysis

For our first set of research questions, we take an analytical approach similar to Leong and colleagues’ paper^35^, in which we explore whether inter-brain synchrony is stronger overall during moments of mutual gaze. Although this was not a replication study, we attempted to translate previous structured experimental designs into a more naturalistic context. For these analyses, we preselected frequency bands and electrodes of interest, based on the findings of Leong and colleagues^35^. Consistent with these findings we expected to observe greater inter-brain synchrony, in Theta and Alpha during all moments of mutual versus non-mutual gaze. Inter-brain synchrony was measured using PLV and PDC computed over EEG data, averaged over central electrodes (C3 and C4).

### Inter-brain event-locked analysis

Investigating inter-brain synchrony during social interactions as a time invariant phenomenon makes it difficult to understand the underlying mechanisms^39^. Therefore, for our second set of research questions, we wanted to explore how inter-brain synchrony changes around gaze onsets. We compared inter-brain synchrony values around sender and receiver mutual versus non-mutual gaze onsets. We expected to observe greater inter-brain synchrony (measured using PLV and PDC, in frequencies 2–18 Hz, over occipital electrodes) around mutual versus non-mutual gaze onsets. For all inter-brain analyses, we used one measure of concurrent (PLV) and one measure of sequential (PDC) synchrony in order to try to better understand how sender and receiver dynamics influence inter-brain synchrony.

### Intra-brain event locked analysis

It has been suggested that one mechanism that might mediate changes in inter-brain synchrony is mutual phase resetting in response to the onset of mutual gaze^27,35^. For our third set of research questions, we wanted to investigate if changes in inter-brain synchrony around mutual gaze onsets also cooccurred with changes in intra-brain amplitude/ power and phase as this might help to understand, mechanistically how inter-brain synchrony might develop. To examine this, we first looked at ERPs and inter-trial coherence (ITC) around gaze onsets: comparing sender and receiver mutual and non-mutual gaze onsets. Inter-trial coherence measures the consistency in phase angles over trials/ time at a single given electrode. By measuring how the consistency of phase angles vary over time one is able to observe whether the onset of some event/ stimulus associates with an abrupt shift in phase (increase in phase coherence). An increase in phase coherence following some event (relative to some baseline or peristimulus period) is often taken as evidence of phase resetting^67^. In comparison PLV measures the consistency in phase angle differences between two electrodes. Based on previous findings (e.g.^8^) we expected to observe significant ERPs and increases in ITC relative to both sender and receiver mutual and non-mutual gaze onsets. Although during receiver non-mutual gaze onsets only, the receiver was looking at an object and not at their partner’s face, research has shown that humans are highly sensitive to eye gaze in their peripheral vision^68^. ERPs and ITC were first assessed against a baseline to test whether there was a significant event-locked neural response relative to both sender and receiver mutual and non-mutual gaze onsets. We then examined whether ERPs and event locked ITC was greater around sender and receiver mutual versus non-mutual gaze onsets. We expected to observe larger ERP amplitudes, and greater ITC (in frequencies 2–18 Hz, over occipital electrodes) around mutual versus non-mutual gaze onsets. There is reasonable evidence from screen-based studies to suggest that the responses we might observe following mutual and non-mutual gaze onsets could occur immediately and transiently (within the first 600 ms). However as this is the first study of its kind to measure ERPs and phase resetting around naturally occurring onsets of parent-infant mutual gaze we did not assume the mechanisms involved in screen-based task would necessarily be the same as in real interactions. Therefore, we did not rely heavily on predefined timescales for our analyses, rather we adopted procedures (non-parametric cluster-based permutation testing) that would be sensitive to effects over any time points within the data.

Overall, through these analyses we wanted to explore three questions; (a) do we observe above chance inter-brain synchrony during mutual gaze/around mutual gaze onsets during free-flowing natural parent-infant social interactions. (B) do we observe phase resetting in parents and infants relative to mutual gaze onsets in natural contexts. (C) if we observe above chance parent-infant inter-brain synchrony and phase resetting around mutual gaze onsets, are they linked.

## Methods

### Ethics statement

This study was approved by the Psychology Research Ethics Committee at the University of East London and all research was performed in accordance with the relevant guidelines/regulations. Informed consent from all subjects was obtained. Informed consent from the subjects illustrated in Fig. 1 and/or their legal guardians for publication of identifying information/images in an online open-access publication was obtained. Participants were given a £50 shopping voucher for taking part in the project.

### Participants

Of the 90 infants we tested for this study, 21 contributed no data at all, 6 contributed EEG data that was too noisy even after data cleaning and 4 were lost due to human error, e.g., failed synchronisation triggers. We also excluded all participants with fewer than 5 gaze onsets, leading to an additional 4 datasets being excluded. The final sample contained 55 healthy (23 F), *M* = 12.2-month-old (*SD* = 1.47) infants, that participated in the study along with their mothers.

Informed consent from the subjects illustrated in Fig. 1 and/or their legal guardians for publication of identifying information/images in an online open-access publication was obtained.

### Power calculations

For the non-event locked analysis, as this analysis were based on previous findings, we estimated the required sample size to observe a difference between the two groups (as a product of gaze type), using the G*power tool^69^. For this, we used data from Leong et al.^35^ as an estimator of the expected effect size for the analysis of non-event locked synchrony (${r}^{2}=$ 0.332). Based on an Alpha level of 0.05, in our sample size (*N* = 55) we had a > 99% chance of observing an effect of gaze type on inter-brain synchrony of the magnitude observed in previous work.

### Experimental set-up and procedure

Infants were positioned immediately in front of a table in a highchair. Adults were positioned on the opposite side of the 65 cm-wide table, facing the infant. Adults were asked to stage a ‘three-way conversation’ between the infant and a small hand puppet and to try to spend an equal amount of time looking at the puppet and the infant. Dual EEG was continuously acquired from the parents and infants for the approx. 5 min duration of the play session (M = 386.1, SD = 123.9 s).

### Behavioural data

Video recordings were made using Canon LEGRIA HF R806 camcorders recording at 50fps positioned next to the infant and parent respectively. Video recordings of the play sessions were coded offline, frame by frame, at 50 fps. This equates to a maximum temporal accuracy of ~ 20 ms. Coding of the infant’s and adult’s gaze was performed by two independent coders. Cohen’s kappa between coders was > 85%, which is high^70^. Instances of mutual gaze were defined through post hoc synchronisation of the parents’ and infants’ gaze time series, by identifying moments when both the parent and infant were looking at each other (specifically ‘partner’ gaze was defined as gaze towards a partner’s eyes). EEG was time-locked to the behavioural data offline based on the video coding using synchronized LED and TTL pulses. To verify the synchronisation, we manually identified blinks in the behavioral data and looked to see if this matched the timing of the blinks in the EEG data.

### EEG data acquisition

EEG signals were obtained using a dual 32-channel Biosemi system (10–20 standard layout), recorded at 512 Hz with no online filtering using the Actiview software.

### EEG artifact rejection and pre-processing

A fully automatic artifact rejection procedure was adopted, following procedures from commonly used toolboxes for EEG pre-processing in adults^71,72^ and infants^73,74^. Full details of the pre-processing procedures can be found in^64^. In brief the data was filtered between 1 and 20 Hz and re-referenced to a robust average reference. Then we interpolated noisy channels based on correlation; if a channel had a lower than 0.7 correlation to its robust estimate (average of other channels) then it was removed. The mean number of channels interpolated was 3.9 (*SD* = 2.1) for infants and 3.9 (*SD* = 4.4) for adults. Then for the infant data only we removed sections from the continuous data in which the majority of channels contained extremely high-power values. Data was rejected in a sliding 1 s epoch and based on the percentage of channels (set here at 70% of channels) that exceeded 5 standard deviations of the mean EEG power over all channels. For example, if more than 70% of channels in each 1-s epoch exceeded 5 times the standard deviation of the mean power for all channels then this epoch is marked for rejection. We found that for adults this step was primarily removing activity that could be removed with ICA (e.g., eye movement artifact) without removing entire sections of the data. The average amount of continuous data removed was 11.9% (*SD* = 14.6%) for infants. Finally, we used ICA to remove additional artifacts.

Careful attention was paid to artifact and the amount of variability in the data throughout. In the supplementary materials (see SM 8) we report the results of standard measures of EEG data quality^75^. Here we estimated the standard error of mean over trials (for the occipital electrode cluster used in our analyses) for each of the ERP components we investigated the effects of gaze type (mutual vs non-mutual) over. Including universal measures like these enables fast and easy comparison between studies and allows the overall quality of the data to be readily assessed. We paid particular attention to eye movement artifact. In previous work we designed a system for automatically identifying and removing artifactual ICA components in infant EEG^64^. The automated system was shown to remove most but not all eye movement related artifact time-locked to saccades. Therefore, we cannot entirely rule out that some of the activity in the sender brains relative to sender mutual and non-mutual gaze onsets is an artifact of the gaze shift.

### Time frequency analysis-extracting power and phase

Time–frequency power and phase was extracted via complex Morlet wavelet convolution. The wavelets increased from 2 to 18 Hz in 17 linearly spaced steps and the number of cycles increased from 3 to 10 cycles logarithmically (this approach is generally recommended; see Cohen^76^, chapter 13).

For all non-event locked analyses presented here, frequency bands were selected based on the bands commonly used in infant research: Theta (3–6 Hz) and Alpha (6–9 Hz)^35,77–80^.

## Analysis

### Inter-brain non-event locked analysis—overview

The aim of the analyses in Section "Inter-brain non-event locked analysis—overview" was to investigate our first research question, i.e., do we observe above chance inter-brain synchrony during mutual gaze during free-flowing natural parent-infant social interactions. We defined mutual gaze as times when both the adult and the infant were looking at each other. Non-mutual gaze was defined as times when the infant was looking at the adult and the adult was not looking at the infant (or vice versa). For all non-event locked analyses, EEG was time-locked to the onset of gaze, and the length of the epoch extracted equated to the duration of the look. The average look durations were 2.7 s (SD = 0.9 s) for mutual and 1.6 s (SD = 0.5 s) for non-mutual gaze. All epochs were then concatenated. The mean amount of continuous data available for analysis was 66.1 s (SD = 41.5 s) for mutual and 28.3 s (SD = 17.9 s) for non-mutual gaze. Because ITC (see Cohen^76^, chapter 19) and PDC are sensitive to the amount of available data, we normalised the amount of data present per condition for each participant by identifying which gaze type had the lower number of continuous data samples (n), and re-sampling data from the other gaze type condition, taking 1:n data samples.

#### Inter-brain non-event locked analysis—PDC

Partial directed coherence (PDC) is based on the principles of Granger^81^. It provides information of the extent to which one times series predicts another. PDC is calculated from coefficients of autoregressive modelling according to:

1 $$PDC = \left( {\frac{{A_{xy} \left( f \right)}}{{\sqrt {a_{y}^{*} \left( f \right)} \cdot a_{x} \left( f \right)}}} \right),$$

where $A_{xy} \left( f \right)$ is the spectral representation of bivariate model coefficients and $a_{y}$ and $a_{x}$ are the spectral model coefficient from the univariate autoregressive model fit. Based on previous literature^35^ we chose to compute PDC in 1-s non-overlapping sliding window. We estimated the model order for each segment using Bayesian information criteria (BIC). Model order values were then averaged for all segments. The result was a model order of 5, the same as used by Leong and colleagues^35^, which was then used for all segments.

#### Inter-brain non-event locked analysis—PLV

For the non-event locked analyses, the phase locking value (PLV) was calculated within a single trial over a defined temporal window (e.g.,^82^) according to:

2 $$PLV_{t} = \frac{1}{T}\left| {\mathop \sum \limits_{n = 1}^{T} {\text{e}}^{{i\left( {\phi \left( {t,n} \right) - \psi \left( {t,n} \right)} \right)}} } \right|,$$

where T is the number of observations or time samples within the window, $\phi \left(t,n\right)$ is the phase on observation *n*, at time *t*, in channel $\phi$ and $\psi (t,n)$ at channel $\psi$. As with PDC we chose to compute PLV in 1-s non-overlapping sliding window. For the non-event locked analyses, it was not important to have fine grained information about how inter-brain synchrony varied over time. Therefore a 1 s averaged window was appropriate, chosen based on previous research^35^.

#### Inter-brain non-event locked analysis—group level stats for PLV and PDC

Firstly, we assessed whether PLV and PDC values were significant compared to surrogate data. This was done by calculating the PLV/ PDC between randomly paired infant and adult dyads. We generated 1000 random infant-adult pairings in this way. We then compared the group averages of the observed PLV/ PDC values for the real infant-adult pairings against the randomly permuted distributions. Under the null hypothesis that the interbrain PLV/ PDC between infants and adults is a result of their real-time social interaction, we should observe no above chance inter-brain PLV/ PDC between randomly paired infant-adult dyads. P values were generated by first z-scoring the observed PLV values and then by evaluating the z-scored value’s position on a Gaussian probability density using the Matlab function normcdf.m. For this test, we used a Bonferroni correction for multiple comparisons. This was appropriate here as we were only testing over a limited number of predefined frequencies/channels. Following the statistical procedure adopted by Leong and colleagues^35^, differences in PLV and PDC between mutual and non-mutual gaze were assessed using a two-way repeated-measures ANOVA, taking gaze type and frequency as the within levels, using average, over electrodes C3 and C4, infant-to-adult PDC *(I→A)* and adult-to-infant *(A→I)* PDC values, and for Theta and Alpha bands separately. A Tukey–Kramer correction for multiple comparisons was applied.

### Inter-brain event locked analysis

The aim of the analyses in Section "Inter-brain event locked analysis" was also to investigate our first research question, i.e., do we observe above chance inter-brain synchrony around mutual gaze onsets during free-flowing natural parent-infant social interactions, but using a more temporally fine-grained approach than in the non-event locked analysis by measuring time varying changes in inter-brain synchrony around gaze onsets.

#### Inter-brain event locked analysis—group level stats for PLV and PDC

Firstly, we assessed whether PLV and PDC values were significant compared to surrogate data. We compared all observed time–frequency PLV/PDC values relative to sender and receiver mutual and non-mutual gaze onsets against time–frequency PLV/PDC values time-locked to randomly inserted events within the continuous data. Differences between the real and surrogate data were assessed using cluster-based permutations statistics, using an alpha value of 0.025 (see SM Sect. 5 for full details). Secondly, we examined whether PLV/PDC values were greater for sender/receiver mutual versus non-mutual gaze onsets. This was similarly assessed over all time–frequency points using a cluster-based permutation procedure, comparing results between different types of gaze onset. Importantly, the amount of eye movement artifact was identical between the sets of results being compared.

### Intra-brain event locked analysis

The aim of the analysis in Section "Intra-brain event locked analysis" was to investigate our second and third research questions, i.e., do we observe phase resetting in parents and infants relative to mutual gaze onsets in natural contexts and if we observe above chance parent-infant inter-brain synchrony and phase resetting around mutual gaze onsets, are they linked. We did this by investigating ERPs and ITC around sender and receiver mutual and non-mutual gaze onsets. Previous research suggests that event-locked face-sensitive neural responses are strongest over parietal/ occipital electrodes^64,83^. Therefore, for our event locked analysis we chose to focus on averaged data from a cluster of 5 parietal/occipital electrodes (PO3, PO4, O1, Oz, O2). Additional topoplots are presented in SM 7, which support the choice of electrodes. The EEG signal was divided into events from − 2500 to 2500 ms (t = 0 denotes the onset of gaze). The mean number of events extracted was 21.1 (SD = 10.8) for infant sender/adult receiver and 15.6 (SD = 12.6) for adult sender/infant receiver mutual gaze onsets and 9.9 (SD = 6.2) for infant sender/adult receiver and 28 (SD = 18.3) for adult sender/infant receiver non-mutual gaze onsets. We matched the number of events between gaze types for each participant using the procedure described in Section "Inter-brain non-event locked analysis—overview", above.

#### Intra-brain event locked analysis—Inter trial coherence

Inter trial coherence (ITC) measures the consistency of frequency band-specific phase angles over trials, time-locked to a specific event. The phase coherence value is computed according to:

3 $$ITC = \left. \frac{1}{N} \right|\mathop \sum \limits_{k = 1}^{N} {\text{e}}^{{i\phi \left( {t,k} \right)}} ,$$

where *N *is the number of trials and $\phi \left(t,k\right)$ is the phase on trial *k*, at time *t*.

#### Intra-brain event locked analysis—ERPs

Following previous research^84^ amplitudes of the P1, N290, and P400 ERPs were measured by calculating the change in amplitude between the peak of the component of interest and the peak of the preceding component. Also following previous recent research^84–87^ we used semiautomated and individualised time window selection^88^. Differences in peak amplitude were quantified using the adaptive mean approach. This process involves first identifying the peak latency of the ERP potential on a participant-by-participant basis using a broad time window. Once the peak latency has been identified an average of the activity in a 20 ms window around the peak is then taken (e.g., as described in^89^). We focused on three major components relevant for face/gaze processing: the P1 component, the N170 (commonly N290 in infant EEG^84^) and the P300 (commonly P400 in infant EEG^84^). For the P1 component we used a time window of 0 to 200 ms for adults and 100 to 300 ms for infants. For the N170/ N290 component we used a time window of 100 to 300 ms for adults and 200 to 400 ms for infants. For the P300/P400 component we used a time window of 200 to 500 ms for adults and 300 to 600 ms for infants. These were selected based on visual inspection of the averaged waveforms. All ERP data were baseline corrected using data from the time window − 1000 to − 700 ms pre-gaze onset.

#### Intra-brain event locked analysis—group level stats for ERPs

To test whether the onset of gaze associated with significant changes in amplitude relative to both sender and receiver mutual and non-mutual gaze onsets we again used nonparametric permutation testing. Here the null hypothesis was that the timing of the gaze onset (e.g., time 0) is unrelated to the observed neural response within the time window examined. To test this, we randomly permuted the time points of the ERPs and took the average (separately around the maximum and minimum points) of the permuted ERP in the time window 0 to + 500 ms. This procedure was then repeated 1000 times, randomising and reshuffling the ERP on each permutation. Finally, an estimate of the permutation p-value was calculated using the z-scoring procedure outlined in “Inter-brain non-event locked analysis—group level stats for PLV and PDC” Here we used cluster-based permutation statistics, using an Alpha value of 0.025 to correct for multiple comparisons. The results are reported in Section "Intra-brain analysis—ERPs".

#### Intra-brain event locked analysis—group level stats for ITC

Firstly, we assessed whether ITC values were significant compared to surrogate data. We compared the observed time–frequency ITC values relative to sender and receiver mutual and non-mutual gaze onsets against time–frequency ITC values time-locked to randomly inserted events within the continuous data. The number of random events was matched based on the number of real gaze onsets available for each participant. Differences between the real and surrogate data were assessed using cluster-based permutation statistics, using an Alpha value of 0.05. We then compared differences between sender and receiver mutual versus non-mutual gaze in ITC using the same cluster-based permutation procedure see Section “Inter-brain event locked analysis—group level stats for PLV and PDC”.

### Bayes factor analyses

To further test the significance of gaze type on inter-brain synchrony we calculated the Bayes Factor. The Bayes factor is a measure of evidence in favour of one statistical model compared to another^90^. It can be advantages to compute as it can quantify relative evidence for both alternative and null hypothesis^91^ compared with standard *p*-values which only indicate the likelihood that the null hypothesis is true. It is defined through the ratio between the likelihood of the data under the null hypothesis and the likelihood of the data under the alternative hypothesis^92^. The greater the Bayes factor the greater the evidence for the alternative hypothesis. We also computed the Bayes factor (BF01) for the absence of an effect (null hypothesis). Results of the Bayes factor analyses are reported in Sections "Inter-brain non-event locked analysis—PLV and PDC" and "Inter-brain event locked analysis—PLV and PDC".

## Results

Before turning to our main research questions, we first calculated descriptive statistics to show how gaze onsets were distributed in our sample (Fig. 2). These results indicate that infants spent, in total, 34%/31%/35% of the total interaction looking to partner/puppet/inattentive (Fig. 2A). Parents spent 62%/31%/7% of the total interaction looking to partner/puppet/inattentive (Fig. 2D). Overall, mutual gaze periods were longer than non-mutual gaze periods (Fig. 2C). The distributions of sender and receiver mutual and non-mutual gaze onsets are given within Fig. 2 (E shows distributions of infant sender/adult receiver mutual and non-mutual gaze onsets and F shows distributions of adult sender/infant receiver mutual and non-mutual gaze onsets). Differences in the frequency of gaze onsets to different types of gaze were normalised using the procedures described above.

### Inter-brain non-event locked analysis—PLV and PDC

Section "Inter-brain non-event locked analysis—PLV and PDC" reports the results of our analyses aimed at investigating our first research question, i.e., do we observe above chance inter-brain synchrony during mutual gaze during free-flowing natural parent-infant social interactions. Here to investigate the relationship between inter-brain synchrony and mutual gaze, we first computed the mean PLV and PDC values across all mutual and non-mutual gaze periods in Theta and Alpha bands separately. We looked at whether PLV and PDC values were significantly greater than baseline, and then compared these values for mutual versus non-mutual gaze. Based on our previous research^35^ we focused on activity over vertex electrodes (C3 and C4).

**Figure 3:**
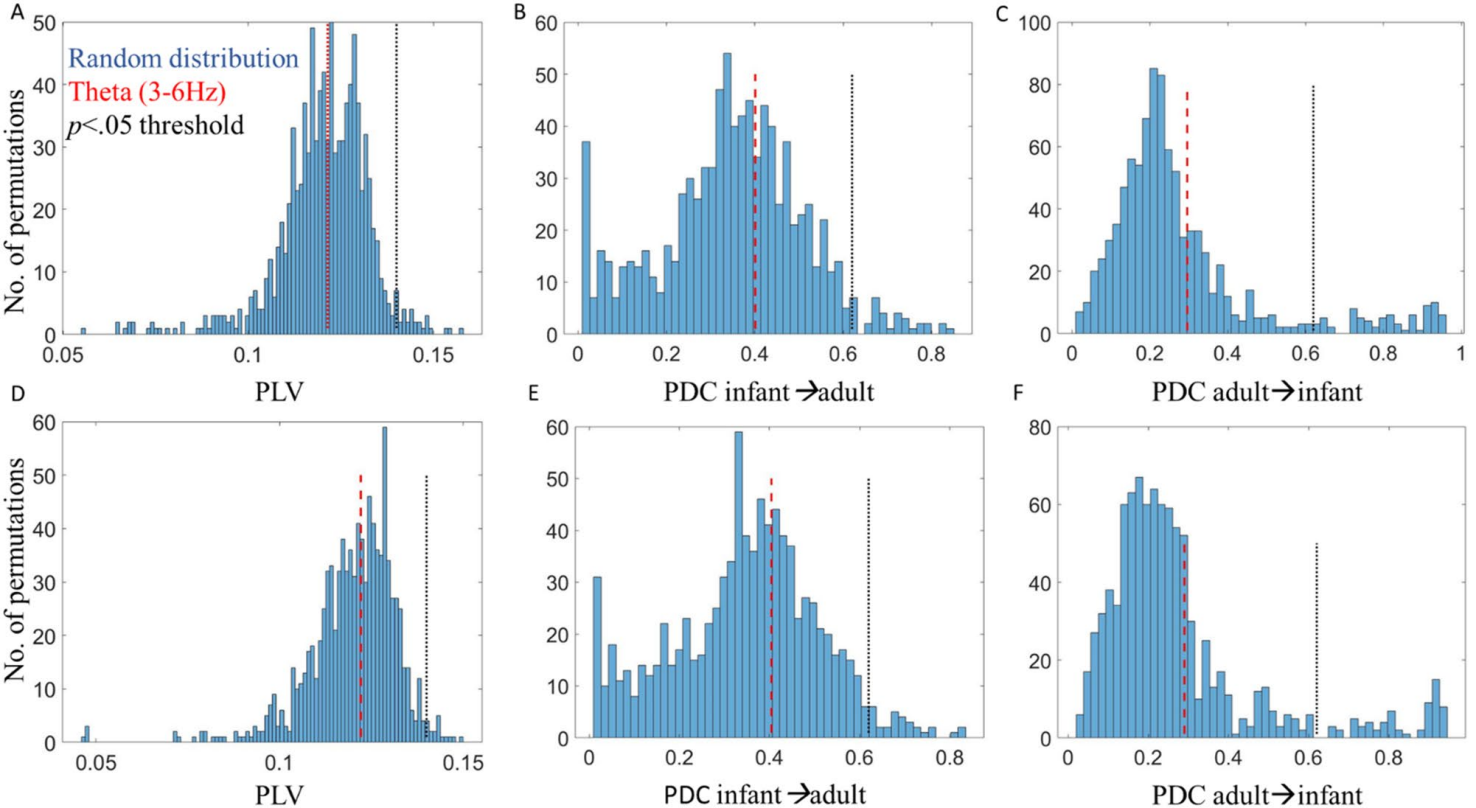
Results of permutation tests for non-event locked inter-brain synchrony analysis. (**A**) Distribution of random pair infant-adult PLV values compared to real pair infant-adult PLV values in Theta for mutual gaze. (**B**) Distribution of random pair infant→adult PDC values compared to real pair infant→adult PDC values in Theta for mutual gaze. (**C**) Distribution of random pair adult→infant PDC values compared to real pair adult→infant PDC values in Theta for mutual gaze. (**D**) Distribution of random pair infant-adult PLV values compared to real pair infant-adult PLV values in Theta for non-mutual gaze. (**E**) Distribution of random pair infant→adult PDC values compared to real pair infant→adult PDC values in Theta for non-mutual gaze. (**F**) Distribution of random pair infant→adult PDC values compared to real pair adult→infant PDC values in Theta for non-mutual gaze. Red dashed lines indicate averaged observed values in Theta, black dotted lines indicate the threshold for *p* < 0.05.

**Figure 4:**
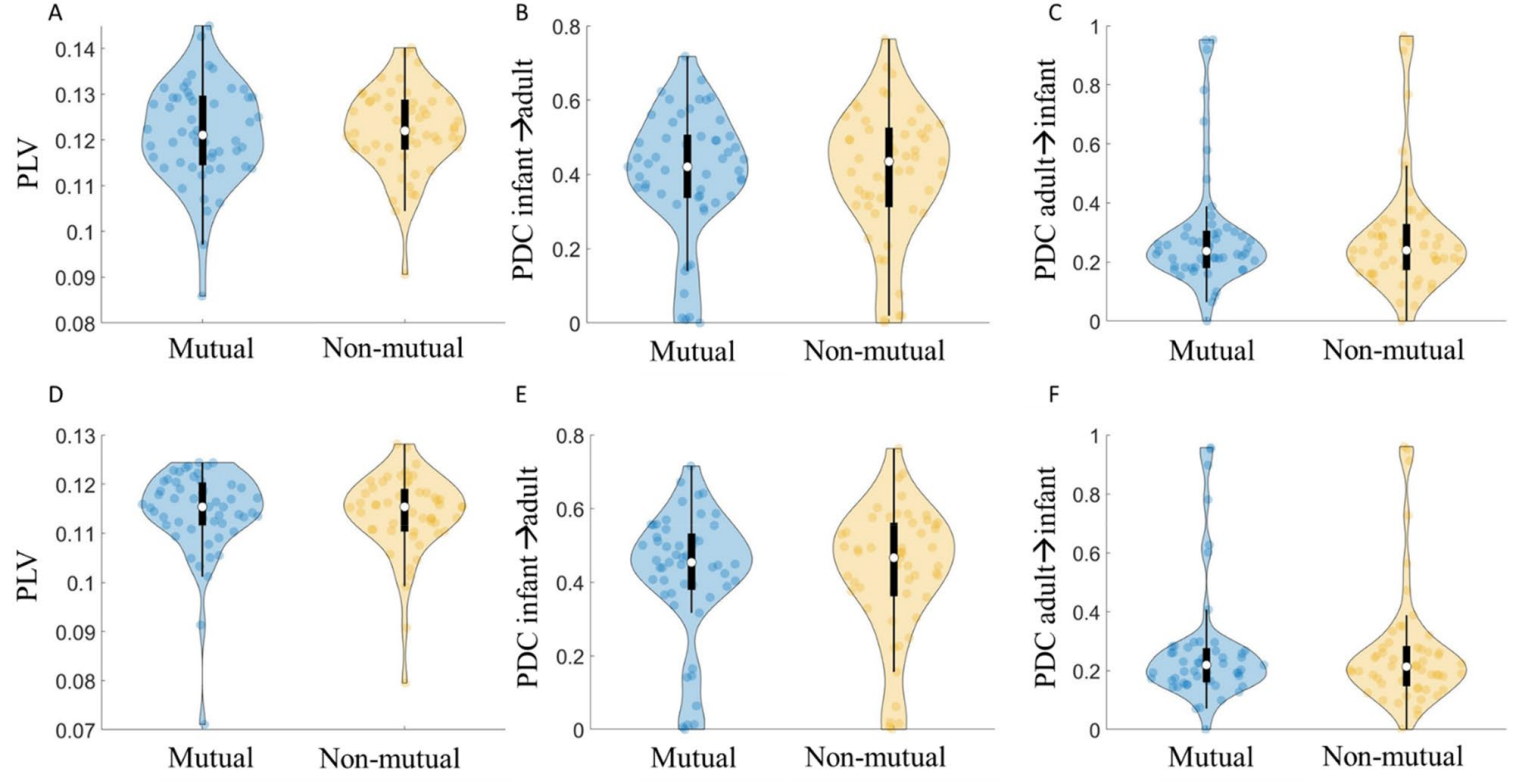
Results of non-event locked inter-brain synchrony analysis. (**A**) Infant-adult PLV during mutual/non-mutual gaze in Theta. (**B**) Infant→adult PDC during mutual/non-mutual gaze in Theta. (**C**) Adult→infant PDC during mutual/non-mutual gaze in Theta. (**D**) Infant-adult PLV during mutual/non-mutual gaze in Alpha. (**E**) Infant-adult PLV during mutual/non-mutual gaze in Alpha. (**F**) Adult→infant PDC during mutual / non-mutual gaze in Alpha. Violin plots show the distribution of the data with an inset boxplot. Each point corresponds to the average PLV/PDC value of a dyad.

Figures 3 and 4 show the results of the inter-brain non-event locked analysis. We first tested whether PLV and PDC values significantly exceeded baseline values. The results of the permutation analysis indicated that PLV values did not significantly exceed baseline values: in theta (3–6 Hz) for mutual (*p* = 0.53) or non-mutual gaze (*p* = 0.56). Further, infant-to-adult PDC *(Infant*→*Adult)* and adult-to-infant *(Adult*→*Infant)* did not significantly exceed baseline values for mutual (*p* = 0.63/*p* = 0.55) or non-mutual gaze (*p* = 0.64/*p* = 0.54); or in alpha (6–9 Hz) for mutual (*p* = 0.57) or non-mutual gaze (*p* = 0.59). Further, infant-to-adult PDC *(Infant*→*Adult)* and adult-to-infant *(Adult*→*Infant)* did not significantly exceed baseline values for mutual (*p* = 0.59/*p* = 0.53) or non-mutual gaze (*p* = 0.61/*p* = 0.52).

We then wanted to test whether PLV and PDC values were greater for mutual than non-mutual gaze. As described in Section "Inter-brain non-event locked analysis—group level stats for PLV and PDC", we then conducted repeated measures (RM) ANOVAs using average indices (over C3 and C4), taking frequency (2 levels) and gaze type (mutual vs non-mutual; 2 levels) as within-subjects factors. The results of the ANOVA indicated no statistically significant differences in PLV,* F*(1, 55) = 0.04, *p* = 0.84, or PDC; for infant to adult (*Infant*→*Adult*) influences, *F*(1, 55) = 0.18, *p* = 0.68 or adult to infant (*Adult*→*Infant*) influences, *F*(1, 55) = 0.50, *p* = 0.48, between mutual and non-mutual gaze. The results did indicate a significant effect of frequency (i.e., more synchrony in Theta than Alpha) for PLV, *F*(1, 55) = 47.33, *p* < 0.01 and PDC; *Infant*→*Adult*, *F*(1, 55) = 41.2, *p* < 0.01 and *Adult*→*Infant*, *F*(1, 55) = 138.84, *p* < 0.01). These results are summarised in Fig. 4. Note these are uncorrected p values.

To further test the significance of gaze type on non-event locked synchrony (PLV and PDC) we calculated the Bayes Factor (BF) at the group level for both Theta and Alpha and (for PDC) including both directions of influence. We calculated ${BF}_{10}$ = ${p(D|H}_{1})$/${p(D|H}_{0})$, where $D$ represents the data and ${H}_{1}$ and ${H}_{0}$ of the alternative and the null hypothesis respectively, using functionality from the bayesFactor toolbox^92^. The BF10 tests for the presence of an effect. For all tests, the BF10 was between 1/3 and 1/10 and non-significant indicating moderate evidence^93^ for the null hypothesis (that there is no difference between mutual and non-mutual gaze). We also converted these scores to the Bayes Factor for the absence of an effect (BF01), confirming that there was moderate to strong evidence that there was no difference between the groups. Results of our Bayes Factor analyses are given in full in SM Sect. 6.

To summarise the results of the non-event locked analyses, suggest that mutual gaze does not associate with inter-brain synchrony in this dataset and using this paradigm since (a) the inter-brain synchrony for either gaze type isn’t above shuffled baseline data and (b) isn’t different from a specifically selected other condition (non-mutual gaze).

### Inter-brain event locked analysis—PLV and PDC

**Figure 5:**
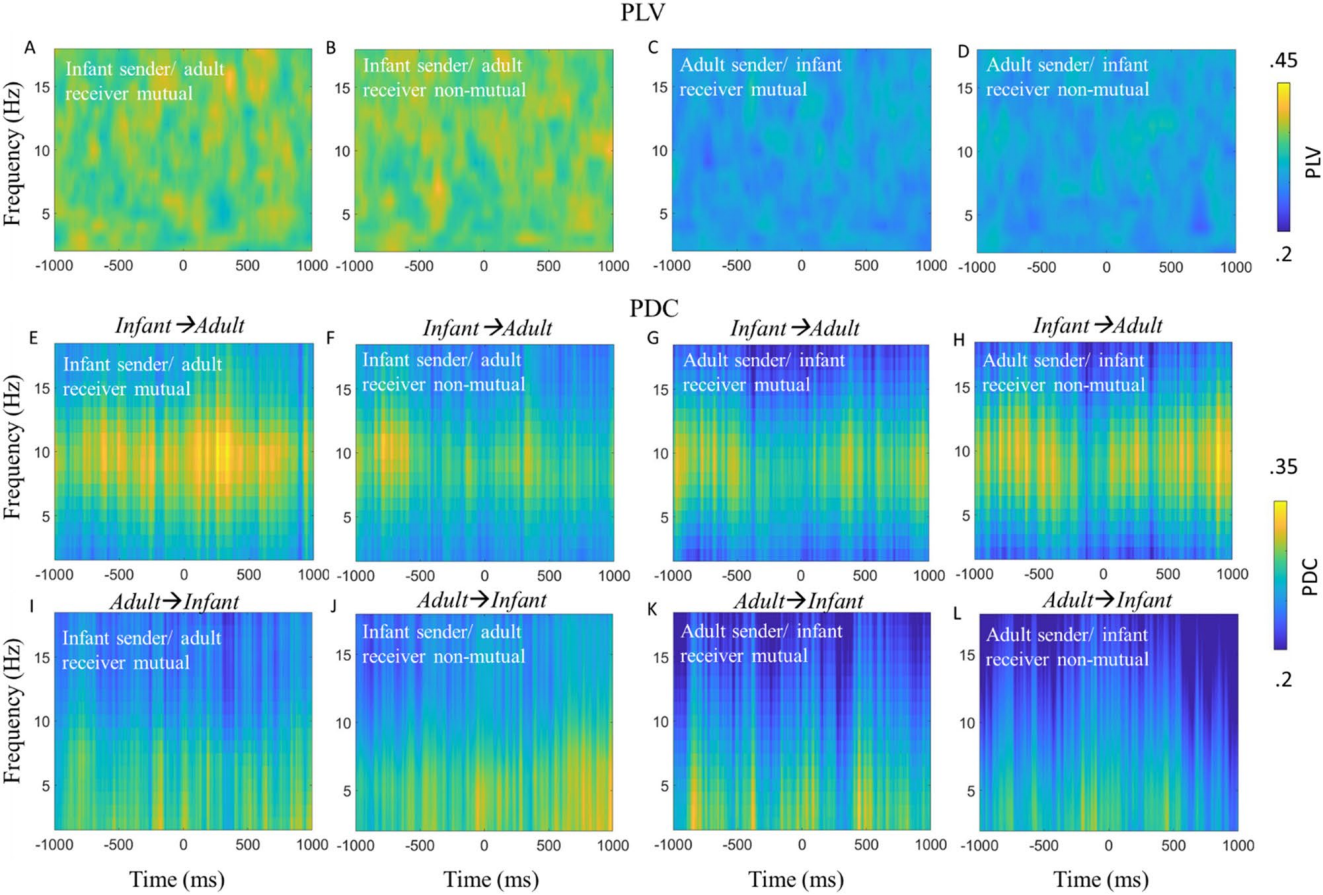
Infant-caregiver inter-brain synchrony time-locked to naturally occurring mutual and non-mutual gaze onsets. (**A**) PLV relative to infant sender/adult receiver mutual gaze onsets. (**B**) PLV relative to onsets of infant sender/adult receiver looks to non-mutual gaze. (**C**) PLV relative to adult sender/infant receiver mutual gaze onsets. (**D**) PLV relative to adult sender/infant receiver non-mutual gaze onsets. (**E**) Infant→Adult PDC relative to infant sender/adult receiver mutual gaze onsets. (**F**) Infant→Adult PDC relative to infant sender/adult receiver non-mutual gaze onsets. (**G**) Infant→Adult PDC relative to adult sender/infant receiver mutual gaze onsets. (**H**) Infant→Adult PDC relative to adult sender/infant receiver non-mutual gaze onsets. (**I**) Adult→Infant PDC relative to infant sender/adult receiver mutual gaze onsets. (**J**) Adult→Infant PDC relative to infant sender/adult receiver non-mutual gaze onsets. (**K**) Adult→Infant PDC relative to adult sender/infant receiver mutual gaze onsets. (**L**) Adult→Infant PDC relative to adult sender/infant receiver non-mutual gaze onsets. No significant differences were identified.

Section "Inter-brain event locked analysis—PLV and PDC" reports the results of our analyses aimed at investigating our first research question, i.e., do we observe above chance inter-brain synchrony around mutual gaze onsets during free-flowing natural parent-infant social interactions, but using a more temporally fine-grained approach than in the non-event locked analysis by measuring time varying changes in inter-brain synchrony around gaze onsets. To do this we investigated whether onsets of mutual gaze associated with changes in inter-brain synchrony and examined the sender-receiver dynamics that might contribute to this. To do this we conducted event-locked analyses with respect to gaze onsets (presented in Fig. 5). We first examined whether PLV and PDC values were significantly greater than baseline around gaze onsets, and then compared these values between mutual versus non-mutual gaze onsets.

We first tested whether PLV and PDC values over occipital electrodes and in the 2–18 Hz range significantly exceeded baseline values generated from a permutation procedure for infant sender/ adult receiver and adult sender/ infant receiver looks to mutual and non-mutual gaze. The result of the permutation analysis indicated that event locked PLV and PDC values around mutual and non-mutual gaze onsets were not significantly different from baseline values (see SM Sect. 5 for full details). Therefore, we failed to reject the null hypothesis that there are no changes in PLV and PDC that are time-locked to gaze onsets.

We also observed no statistically significant differences for the effect of gaze type (e.g., mutual vs non-mutual). Therefore, we failed to reject the null hypothesis that there was no difference in PLV and PDC between looks to mutual or looks to non-mutual gaze (see SM Sect. 5 for full details of permutation statistics).

To further test the significance of gaze type on event locked inter-brain synchrony (PLV and PDC) we calculated the Bayes Factor at the group level for both Theta and Alpha and (for PDC) including both directions of influence. For all tests, the BF10 was between 1/3 and 1/10 and non-significant indicating moderate evidence^94^ for the null hypothesis (that there is no difference between mutual and non-mutual gaze). We also converted these scores to the Bayes Factor for the absence of an effect (BF01), confirming that there was moderate to strong evidence that there was no difference between the groups. Results of our Bayes Factor analyses are given in full in SM Sect. 6.

### Intra-brain analysis—ERPs

**Figure 6:**
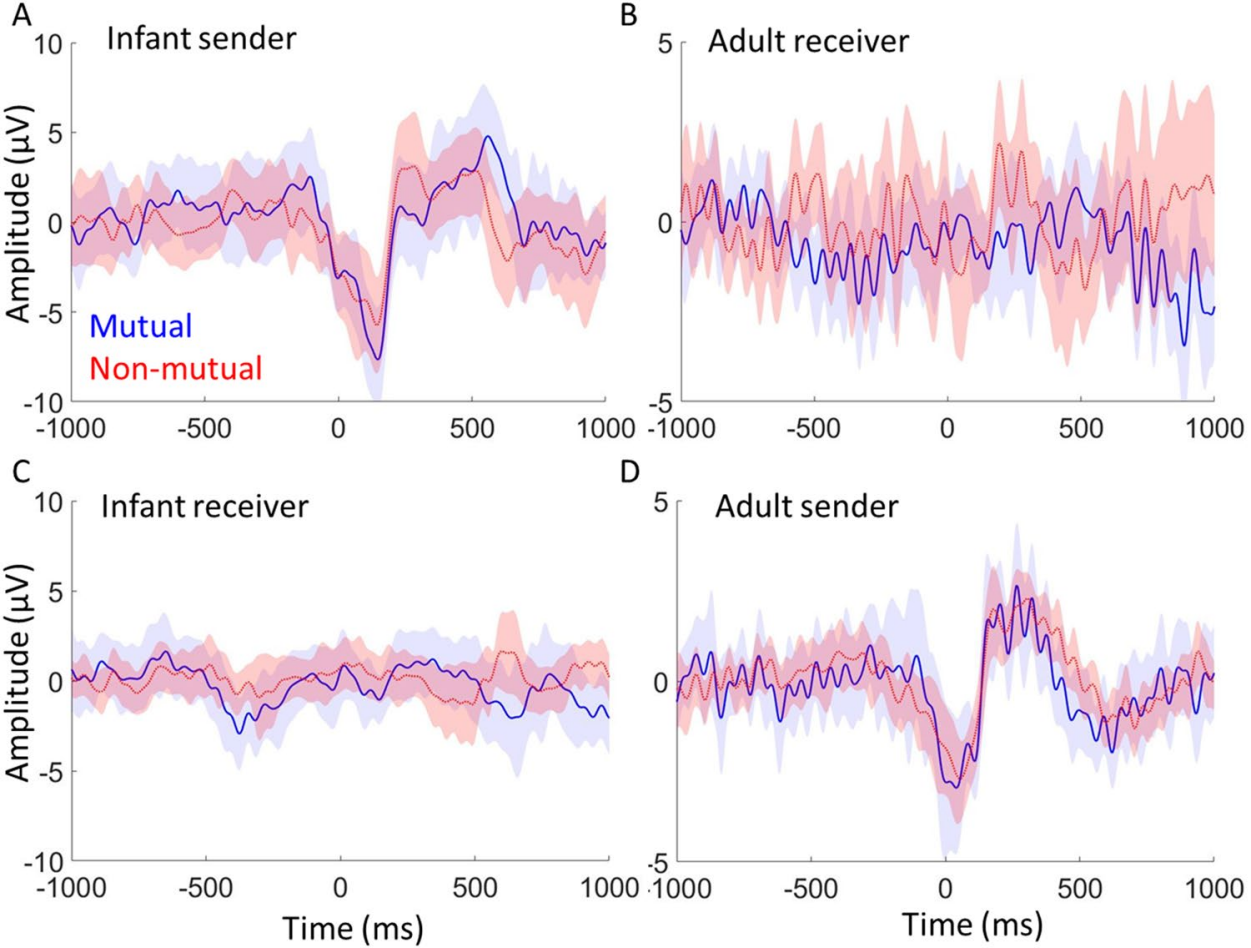
Event-related potentials time-locked to naturally occurring mutual and non-mutual gaze onsets. (**A**) Infant ERP relative to onsets of infant sender mutual gaze and non-mutual gaze onsets. (**B**) Adult ERP relative to adult receiver mutual and non-mutual gaze onsets. (**C**) Infant ERP relative to onsets of infant receiver mutual and non-mutual gaze onsets. (**D**) Adult ERP relative to adult sender mutual and non-mutual gaze onsets. For each the shaded area indicates 95% confidence intervals; thicker lines indicate grand average waveforms. Additional topoplots can be found in SM 7.

Section "Intra-brain analysis—ERPs" reports the results of our analyses aimed at investigating our second and third research questions, i.e., do we observe phase resetting in parents and infants relative to mutual gaze onsets in natural contexts and if we observe above chance parent-infant inter-brain synchrony and phase resetting around mutual gaze onsets, are they linked. Here, we wanted to examine intra-brain sender/receiver dynamics around mutual gaze. To do this we compared ERPs between sender and receiver mutual versus non-mutual gaze onsets in both infants (Fig. 6A-infant sender mutual and non-mutual and Fig. 6C infant receiver mutual and non-mutual) and adults (Fig. 6D- adult sender mutual and non-mutual and Fig. 6B adult receiver mutual and non-mutual).

Figure 6 shows the results of the intra-brain ERP analysis, comparing sender and receiver mutual and non-mutual gaze onsets. We first tested whether ERP values for sender and receiver mutual and non-mutual gaze onsets significantly exceeded baseline values generated from a permutation procedure (see Section "Intra-brain event locked analysis—group level stats for ERPs"). The permutation analysis indicated that ERP amplitudes (this is just looking at whether there is a positive peak in the 0–500 ms time window) in the post gaze onset window were significantly higher than baseline for sender mutual (*p* < 0.01 for both infants and adults) and non-mutual (*p* < 0.01 for both infants and adults) gaze onsets, in both infants and parents, but not for receiver mutual (*p* = ${P}_{z}$*,* for infants and *p* = 0.2, for adults) or non-mutual (*p* = ${P}_{z}$*,* for infants and *p* = 0.6, for adults) gaze onsets in either parents or infants.

We then compared ERP amplitudes between mutual and non-mutual gaze onsets. As the ERP amplitudes were non-significant over baseline, relative to receiver mutual and non-mutual gaze onsets we focused our comparison on sender mutual versus non-mutual gaze onsets. The results of the paired samples t-test indicated no statistically significant differences after correcting for multiple comparisons. This was consistent for all three components; P1 (*p* = 0.66 for the infant data and *p* = 0.04 for the adult data; uncorrected p-values), N170/N290 (p = 0.61 for infants and *p* = 0.45 for adults) and P300/P400 (*p* = 0.21 for infants, *p* = 0.59 in adults).

Throughout the event locked analyses careful attention was paid to what activity reflected genuine neural responses and what was related to artifact. To investigate this in more detail we performed additional analyses (see SM Sect. 2) in which we compared the senders’ neural responses pre and post artifact cleaning and compared activity over frontal versus occipital electrodes. The results of this analysis suggested that it is unlikely that these findings are driven by the eye movement artifact itself, but rather the resulting neural response. In order to further test the sensitivity of our paradigm, we also compared sender neural responses between looks to object versus looks to partner gaze (see SM Sect. 1). The results of this analysis suggested that our paradigm differentiates neural responses to face versus object looks, consistent with the results from previous ERP studies.

### Intra-brain event locked analysis—ITC

Lastly, we examined the possibility that the onset of mutual gaze could act as synchronisation triggers to concomitantly reset the phase of the sender and receiver’s ongoing neural oscillations.

**Figure 7:**
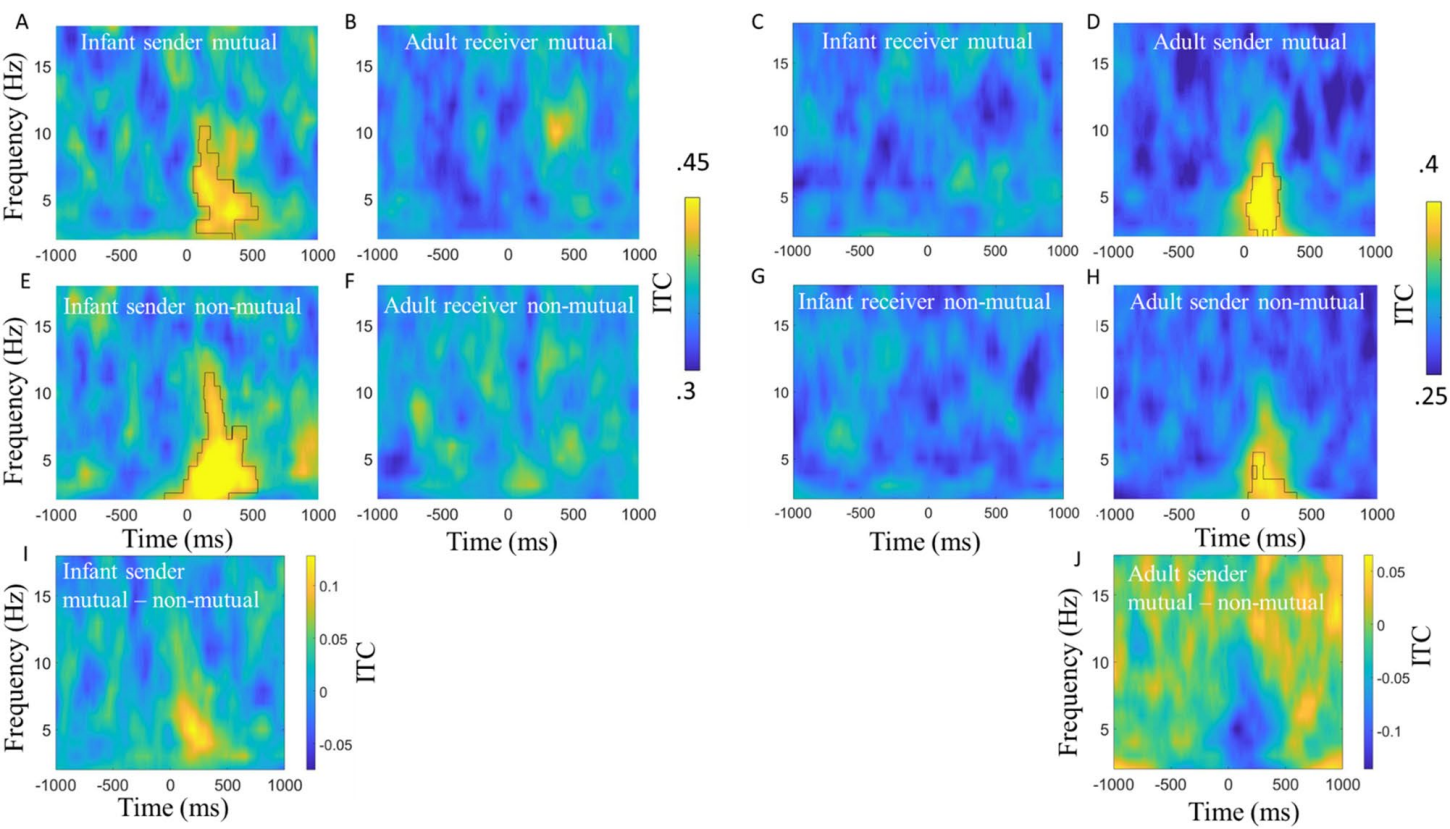
Inter-trial phase coherence time-locked to naturally occurring mutual and non-mutual gaze onsets. (**A**) Infant ITC relative to infant sender mutual gaze onsets. (**B**) Adult ITC relative to adult receiver mutual gaze onsets. (**C**) Infant ITC relative to infant receiver mutual gaze onsets. (**D**) Adult ITC relative to adult sender mutual gaze onsets. (**E**) Infant ITC relative to infant sender non-mutual gaze onsets. (**F**) Adult ITC relative to adult receiver non-mutual gaze onsets. (**G**) Infant ITC relative to infant receiver non-mutual gaze onsets. (**H**) Adult ITC relative to adult sender non-mutual gaze onsets. For all, black borders highlight activity that was significantly greater than baseline after cluster correction for multiple comparisons using *p* = 0.05. (**I**) Difference plot between infant sender mutual versus non-mutual gaze onsets. (**J**) Difference plot between adult sender mutual versus non-mutual gaze onsets. Hotter colours indicate more ITC for mutual versus non-mutual.

Figures 7 shows the results of the event-locked ITC analysis, comparing sender and receiver mutual and non-mutual gaze onsets for infant and adults separately. We first tested whether ITC values, over occipital electrodes, and frequencies 2–18 Hz, significantly exceeded baseline values generated from a permutation. The permutation analysis indicated that ITC in the post-gaze onset window was significantly higher than baseline for sender mutual (Fig. 7A,D) and non-mutual (Fig. 7E,H) gaze onsets, in both parents and infants, but not for receiver mutual (Fig. 7B,C) and non-mutual (Fig. 7F,G) gaze onsets in either parents or infants.

We then wanted to test whether ITC values were greater for mutual versus non-mutual gaze onsets. The results of the cluster-based permutation analysis indicated no statistically significant differences between looks to mutual versus non-mutual gaze in the sender’s brain activity in either parents or infants. The permutation analysis did reveal that ITC in the post gaze onset window was significantly greater relative to infant, but not adult receiver mutual versus non-mutual gaze onsets (peaking in Theta between 0 and 500 ms post gaze onset, full details can be found in SM Sect. 4).

## Discussion

We took dual EEG recordings from parents and infants whilst they engaged in naturalistic free-flowing social interactions. Our data were analysed using cleaning and analysis procedures specially designed for naturalistic dual EEG data^39,64^. Since our analyses suggested that eye movement artifact cannot be completely removed from the EEG, we primarily compared sections of the data that were both identically time-locked to saccades, and therefore contain (presumably) an identical amount of eye movement artifact. The saccades were differentiated by the consequences of the saccade (either the saccade leads to mutual gaze, or not). Furthermore, we also compared activity only in the later parts of the ERP waveform after the first 100 ms, when we were confident that no residual artifact remained.

Overall, through our analyses we wanted to explore three questions; (a) do we get above chance inter-brain synchrony during mutual gaze/around mutual gaze onsets during free-flowing natural parent-infant social interactions. (B) do we get phase resetting in parents and infants relative to mutual gaze onsets in natural contexts. (C) if we get above chance parent-infant inter-brain synchrony and phase resetting around mutual gaze onsets, are they linked. As the result of our analysis indicated that inter-brain synchrony was not greater than chance during mutual gaze/around mutual gaze onsets during natural parent-infant social interactions we were not able to answer question c. The discussion focuses on the analysis conducted to try to address these questions.

### Mutual gaze and inter-brain synchrony

Our first set of research questions explored whether inter-brain synchrony was greater during mutual versus non-mutual gaze. Our results indicated that inter-brain synchrony did not significantly exceed baseline values for either mutual or non-mutual gaze. Further, comparing mutual versus non-mutual gaze, our results indicated that inter-brain synchrony was not greater during mutual versus non-mutual gaze, contrary to what we had hypothesised. These null findings were consistent across both frequencies and both measures of synchrony that we looked at (PLV and PDC), and across both our non-event and event locked analyses.

These results are inconsistent with previous studies that observed greater inter-brain synchrony during continuous (i.e., not relative to specific behaviours/ events within the interaction, but rather looking across all moments of a given behaviour during social interaction) moments of mutual versus non-mutual gaze. For example, in our previous paper we found increased inter-brain synchrony using PDC, in Theta and Alpha, over C3 and C4 electrodes in *N* = 29 *8*-month-olds^35^. In the present study, we measured PDC and PLV across the same frequencies and electrodes in *N* = 55 *12*-month-olds.

Although we followed the same analytical techniques as Leong et al.^35^, we used different pre-processing techniques and a different (less structured, more naturalistic) paradigm, which could explain why our results differ. Firstly, the previous study featured an unfamiliar live adult singing nursery rhymes to an infant. Our present study, in contrast, featured primary caregivers interacting freely with the infant, using a puppet that they held in their hand. Infants’ sensitivity to novel interaction partners is well documented^95–99^. Therefore, one explanation for the positive effects of gaze type on inter-brain synchrony in our previous study could be due to the saliency of mutual gaze in the presence of an unfamiliar adult. To investigate this further we performed the same analysis with data collected from infant-adult dyads. Here the infants interacted with an unfamiliar adult (one of two research assistants). The results of these analyses are reported in full in SM 9, but summarised here, we found consistent with our main analyses of infant-caregiver dyads inter-brain synchrony was not above chance around mutual gaze onsets and did not differ between mutual and non-mutual gaze onsets. Further we found that phase resetting around mutual gaze onsets was strongest for infant and adult sender compared with receiver gaze onsets. Although in this current work we have looked at how our findings generalise across two different dyad pairings (e.g., parent-infant and unfamiliar adult-infant dyads) it will be important for future research to investigate mutual gaze onsets and inter-brain synchrony in other dyad pairings including adults and infants/children of different ages than examined here and also adult-adult dyads. Second, in our previous study, adults continuously sung nursery rhymes to the infants during the interactions, whereas in our present study they talked normally. As sung nursery rhymes are highly periodic^100^ and evidence suggests that infant’s neural activity entrains to the temporal structure of these songs^101,102^ it could be that the regularity of the nursery rhymes introduced an external periodic stimulus into the environment that was driving the inter-brain entrainment^103^. Here, mutual gaze might only enhance or maintain synchrony that is already established, by facilitating shared attention and therefore upregulating attention-enhanced neural synchrony. It will be important for future research to examine inter-brain synchrony in a variety of settings, ranging from very unstructured settings such as those used in the present study to more structured settings in which there are environmental stimuli with more regular and predictable inputs. Our use of a naturalistic puppet play paradigm was both a strength and a weakness. The puppet play paradigm created comparatively more instances of mutual and non-mutual gaze than other naturalistic settings (e.g., joint play with multiple toys). However, one limitation of this is that the puppet may have introduced a separate condition that could have constituted a kind of social intermediary between direct mutual gaze and complete social inattention (or gaze elsewhere in the room). To increase the number of trials in our analyses our non-mutual gaze category encompassed moments when the sender/receiver was either looking to the puppet or elsewhere in the room. Although beyond the scope of the present analyses, it is possible that in addition to mutual gaze, there are at least 3 conditions (gaze to other, gaze to puppet, gaze away elsewhere in the room, and maybe even mutual gaze to puppet) that might represent qualitatively and socially distinct experiences, perhaps evoking distinct neural patterns both intra- and interpersonally. A possibility that future research should explore in more detail. Additionally, to address the possibility that differences in pre-processing procedures could explain why we failed to replicate previous findings we cleaned our data following to the best of our ability the pre-processing procedures outlined in Leong and colleagues’ study^35^. The full results of this are reported in SM 10, but to summarise we found that cleaning the data following the procedures of Leong and colleagues^35^ had no impact on the significance of any of the results of the main paper, ruling out the possibility that differences pre-processing procedures might be the cause of the discrepancy. Finally differences in the ages of infants between the present study (10–12 m) and our previous research (Leong et al.^35^ studied 7.5-month-old infants) could have contributed to differences in results. As the evidence we currently have available suggests that even from birth infant’s brain are sensitive to mutual gaze^8^ it will be important for future research to examine developmental effects on parent-infant inter-brain synchrony during social interaction. Overall, these inconsistencies highlight the likely context-specific and localised nature of inter-brain synchrony, and further emphasise the importance of replication and standard data quality measures^75^ when studying inter-brain dynamics^32^. It will be important for future research to look at how these findings generalise to other groups/dyad pairings and to investigate how associations between mutual gaze and inter-brain synchrony vary over the course of development.

### Phase resetting around gaze onsets

For our second set of research questions, we explored event-locked intra and inter-brain neural responses associated with mutual gaze onsets. Through this, we aimed to test our previously published hypothesis that concomitant phase resetting in the sender and the receiver’s brain at the onset of gaze may drive inter-brain synchrony^27,35^. Overall, the results of our event-locked analyses are inconsistent with this idea. Contrary to our hypothesis, inter-brain synchrony did not significantly exceed baseline values for sender/receiver mutual or non-mutual gaze onsets and was not significantly different between sender or receiver mutual versus non-mutual gaze onsets. Further, whilst we found that sender but not receiver mutual and non-mutual gaze onsets associated with significant increases in ITC and amplitude (ERPs) over baseline, we did not find significant differences between sender or receiver mutual versus non-mutual gaze onsets. We did, however, find evidence for increases in ITC relative to sender mutual and non-mutual gaze onsets (Section “Intra-brain event locked analysis—ITC”); but it is difficult to conclude that this represents phase resetting of brain oscillations. It could also be that changes in event locked amplitude/power create the artifactual appearance of phase synchrony^104^—a fact that the close correspondences we observed between ITC and event-locked changes in amplitude/power (see SM 2) would appear to support.

One possible driver of the sender neural responses could be residual eye movement artifact in our data. In the supplementary analysis (SM. 3) we compare time–frequency power over frontal and occipital electrodes before and after ICA cleaning and report that ICA cleaning removed most, but not all, of the assumed artifactual activity associated with the eye movement- a conclusion consistent with our previous research^64^. This analysis also allowed us to identify that these artifacts are transient (~ 100 ms) and therefore only impacted the initial part of the ERP waveform. After the initial ~ + 150–200 ms we observed ERP components that look very similar to ERPs observed in traditional screen-based tasks (see Fig. S1), with clear P1, N290 and P400 components. For added safety, however, our main analyses were based on comparing sections of the data that are both identically time-locked to saccades, and therefore contain an identical amount of eye movement artifact.

Overall, then, our results challenge the theory that phase resetting around key communicative signals such as mutual gaze is a mechanism through which inter-brain synchrony is achieved. Assuming that inter-brain synchrony according to more recent frameworks^32^ is associated with mutual gaze. This points to the potential importance of other potential drivers of inter-brain synchrony, that future work should investigate in more detail—such as correlated changes in amplitude/ power or changes in oscillatory frequency independent of phase resetting (see^39^ for a detailed discussion), and other more periodic behaviours (e.g., speech^101,102^). Again, it is worth noting here that it will be important for future research to look at how these findings generalise outside of parent- (12 m old) infant dyads.

### Re-examining the importance of ‘receiver’ mutual gaze in infant-caregiver social interaction

Our third aim was to test the hypothesis that infants are highly sensitive to ostensive signals during free-flowing social interactions with their caregivers. A number of influential papers^1,8,9,21,105^ argue that, from shortly after birth, infants’ brains are sensitive to receiving ostensive signals, and that ‘sender’ communicative signals play a key role during naturalistic learning exchanges. However, as we noted these findings have not replicated well in developmental research; for example, Elsabbagh and colleagues^12^ or in research with adults^13–18^.

Contrary to expectations we found robust neural responses only in the senders’ (i.e., the agent initiating the gaze episode) and not in the receivers’ neural responses. This was true both for receiver non-mutual gaze onsets (where receivers were not looking at their partners and thus may have failed to detect their partners’ gaze shift), but also for the receiver-mutual condition (where receivers were directly gazing towards their partner at the time of the gaze shift). Evidence from adult ERP studies in which dynamic changes in gaze are simulated, through the presentation of a series of static images on a screen suggest that human adults are sensitive to ‘dynamic’ changes in gaze^106,107^. However, these simulated changes in gaze are still far from the continuous way that gaze is processed during real social interactions, and it is likely that the effects observed in these studies are largely driven by more low-level properties of the simulation (e.g., retinal stimulation evoked by the presentation of a series of static images) rather than reflecting the actual processing of the gaze shift. When scrambled control images are presented in this same way this produces similar neural responses to those associated with processing simulated changes in gaze^108^. This suggests that whilst these studies do capture some neural mechanisms that are sensitive to moment-to-moment changes in the visual input from our environment, these studies do a poor job of simulating the continuous flow of gaze information that happens during real life social interaction. However, these studies do show some subtle neural sensitivity to changes in gaze orientation that perhaps we were unable to capture with the level of sensitivity afforded in our current approach. This raises basic questions over where, when and under what circumstances changes in a partner’s gaze during free-flowing social interactions impacts the neural activity of the receiver (the person viewing the gaze shift).

Again, one possible explanation for the inconsistencies between previous screen-based tasks and the present study is simply it is just a result of increased artifact through the use of a naturalistic paradigm. However, we note that: (i) our ERPs show a close visual correspondence with ERPs observed in traditional ERP paradigms (see Fig. 6 and Fig. S1 also); ii) the overall measures of EEG data quality we reported show good quality data (see SM Sect. 8); (iii) we did replicate the findings from screen-based ERP research that infants show enhanced ERPs to images of faces versus objects^85–87,109^ (see also SM Sect. 1); We observed statistically greater occipital ERP amplitudes for faces versus objects for the N290 component, but not for P1 or P400 components. Overall, then, we found changes in brain activity only in the person that initiated the episode of mutual gaze (the sender), and no changes in the recipient of the mutual gaze. This conclusion suggests a different account of the dyadic mechanisms involved in the processing of mutual gaze. In contrast to evidence suggesting that mutual gaze involves both infants and adults reciprocally influencing each other’s neural activity towards shared rhythms^35^, we found changes in brain activity only in the person that initiated the episode of mutual gaze (the sender). This suggests that in this context (i.e., parent and 12 m old infant social interactions), mutual gaze processing is more supported through more basic changes at the intra-brain level, which do not specifically affect dyadic neural mechanisms. For example, evidence suggests that eye movements lead to low frequency phase reorganisation in brain structures such as the hippocampus that are deeper than those that can be measured using scalp EEG^110^. Eye movements may create transient increases in neural sensitivity within certain structures within an individual’s brain that support them in processing the new visual information (e.g., mutual gaze)^40^.

## Conclusion

We investigated the possibility that concomitant phase resetting in response to mutual gaze onsets during naturalistic infant-caregiver interactions might be a mechanism through which inter-brain synchrony is established. We found no evidence for changes in inter-brain synchrony around gaze onsets and no evidence to support our previously published suggestion that phase resetting in the sender and the receiver’s brain around mutual gaze onsets may be a mechanism through which inter-brain synchrony arises^27,35^. Further, contrary to our prediction, we found that mutual gaze onsets associated with neural responses in ‘senders’, but not in ‘receivers’ brains. Overall, our study challenges current views on the importance of mutual gaze. It highlights the fact that we need pluralistic approaches to better understand early social cognition. And it highlights the importance of studying how infants perceive communicative signals during naturalistic interactions, and across different real-world contexts.

## Supplementary Information

Supplementary Information.

## Data Availability

The datasets used and/or analysed during the current study available from the corresponding author on reasonable request.
